# Factors Contributing to Time-Wasting Activities among Palestinian Nurses: A Cross-Sectional Study

**DOI:** 10.1155/2024/6480929

**Published:** 2024-02-01

**Authors:** Raj'a Nayef Zyoud

**Affiliations:** Arab American University-Palestine, P.O. Box 240, 13 Zababdeh, Jenin, State of Palestine

## Abstract

**Background:**

Nurses face significant challenges as they attempt to manage an increasing number of complex responsibilities within limited time frames. This article explores the factors contributing to time-wasting behaviors among nurses in Palestine and emphasizes the significance of effective time-management skills in nursing practice.

**Methods:**

Surveys were collected from a total of 714 nurses working in multiple healthcare facilities located in the north of the West Bank, Palestine. An 11-item time-wasting scale was developed and validated. Factors influencing time-wasting behaviors among nurses were then investigated using multiple linear regression in SPSS version 25.

**Results:**

Attending time management courses significantly reduced time-wasting behaviors. Additionally, factors such as age, gender, and educational level did not appear to correlate with time-wasting behaviors. However, workplace, type of organization, and attendance of time management courses did impact nurses' time management skills.

**Conclusion:**

This article underscores the importance of time management skills in nursing practice. Inefficient time management can have detrimental effects on patient care and nursing outcomes. To mitigate these challenges, healthcare institutions and nursing education programs should prioritize time management training for nurses.

## 1. Introduction

Nurses face ongoing challenges as they try to handle a growing number of complex tasks and demanding work conditions within the constraints of limited time [1]. In a mixed-methods study conducted in Palestine, [2], a nurse captured the challenges they encounter while endeavoring to address the requirements of all their patients: *“You cannot give quality when you are overwhelmed with quantity. When I have 15 patients on a ward to take care of on a night shift, I barely have time to distribute the medications, leaving little time to care for patients” other needs.”* According to Saintsing et al. [3], nurses indicated that time limitations constrained their capacity to conduct thorough patient assessments. Around 80% of entry-level nurses acknowledged making errors due to time pressure. Managing time efficiently and avoiding time-wasting activities are therefore essential in the nursing work environment. The lack of time management and organizational skills was also related to inequities in health service provision and distribution [4].

Causes of time-wasting behaviors among nurses include a lack of effective time management skills. This includes not setting short- and long-term goals, failing to organize tasks, engaging in activities that waste time, and lacking knowledge and experience in setting priorities [5, 6].

Consequences of time-wasting behaviors include ineffective work performance, decreased productivity, and the potential for missing essential nursing care tasks [5]. Nurses who wasted their time and energy on social media and low-priority tasks were found to significantly lower their work performance [7]. Not knowing how to set priorities by giving equal attention to major and minor problems can have dire consequences on patients' health and overall nursing outcomes [8].

Numerous studies have consistently demonstrated a detrimental impact of ineffective time management on job performance [9, 10]. Tang and Vandenberghe [11] explained that demands that exceed an individual's available resources contribute to a decline in overall work performance and well-being. Others found that work overload negatively affects performance through increasing stress [10]. A cross-sectional study from Brazil revealed that work-related stresses had the greatest negative impact on job performance while social networks and job autonomy had the greatest positive impact [12].

A study by Knezevic et al. [13] investigated the effects of time management skills on job satisfaction for nurses. The study found that poor time management skills were significantly associated with lower job satisfaction levels among nurses. In a related study, Ozkan & Timbil [14] found that nurses with poor time management skills made more medication errors and missed nursing care activities more frequently than those with good time management skills. The authors recommended that time management training should be included in nursing education to improve patient safety and quality of care.

Similarly, a study by Cleland Woods et al. [15] found that poor time management skills were associated with higher levels of job-related stress among nurses. The authors recommended incorporating time management training into workplace wellness programs to help nurses manage their time more effectively and reduce job-related stress. In conclusion, poor time management skills can have negative effects on nurses' capacity to provide optimal patient care. It is, therefore, essential that time management skills are included as a critical component of nursing education and workplace wellness programs.

Numerous studies, including those conducted by Hamzehkola and Naderi [16], Higazee et al. [17], Ebrahim et al. [18], and Elsabahy et al. [19], have presented sound evidence that time management interventions can enhance the organizational skills of nurses, mitigate work-related stress levels, and ultimately elevate the overall quality of their work. A time management educational program for head nurses in Iran assessed improvements in five essential time management skills (setting goals, setting priorities, time mechanics, time control, and organizing time). The results showed dramatic improvements in these basic skills after the educational intervention [20].

Another quasi-experimental experiment of 60 nurses from all hospital wards in Tehran examined the psychological and social impacts of a one-day educational workshop on strategies to improve time management and avoid wasting nursing time [16]. A pretest was followed by a posttest one month after the workshop. Results showed significant improvements in psychological well-being and trust among staff.

Setting priorities has been defined as the action of assigning precedence in rank with regard to the importance or time for some activities over others [8]. In addition to delaying activities of less importance, prioritization can also eliminate unnecessary tasks which allow nurses to allocate more precious time for patients [16]. These findings underscore the importance of time management skills for nurses. Prioritizing tasks and activities are crucial aspects of time management skills.

It has been noted that new nurses struggle with managing their time and that their abilities to prioritize tasks and minimize time-wasting activities improve with experience [21]. The degree to which this relationship is a function of experience, age, education, or other variables that evolve with time has not been extensively investigated in previous research [22]. Many studies show that time-wasting activities decrease with age, education, experience, and attending time-management courses. Most of the previous research, however, did not adjust for confounding effects in multivariate analyses [23]. The current study aims at exploring the relationships between these background variables and time-wasting behaviors in multivariate models to distinguish the most important factors contributing to time-wasting behaviors.

### 1.1. Principles of Setting Priorities

The fundamental question is how can nurses determine the priority, importance, and urgency of the multitude of tasks they are tasked with? Several time management theories and models can help nurses in managing their time and minimizing time-wasting activities. The items used in the time-wasting scale of this study such as appreciating planning, distinguishing important tasks, and understanding time-wasting activities reflect those principles. Those principles include

1. The Pareto 80/20 rule notes that 80% of outcomes result from 20% of efforts [24]. This principle calls for identifying and focusing on the most impactful tasks, i.e., the ones that yield the highest returns. The 80/20 rule therefore prioritizes the 20% of tasks that produce the best results.
2. The two-minute rule recommends completing small tasks (e.g., those requiring 2 minutes or less) first before embarking on tasks that are complicated or require long durations [25]. This rule helps minimize the accumulation of small tasks and fosters a sense of progress.
3. The effort impact matrix, also known as the PICK Chart, prioritizes tasks based on impact and effort [26]. The impact can be assessed based on financial gain, patient satisfaction, or health improvement. The impact or yield of a task ranges from low to high.
4. The ABC method involves categorizing tasks based on their priority and then completing the highest priority tasks while less urgent tasks can be delayed or delegated [27].
5. Eisenhower matrix categorizes tasks based on their urgency and importance into four quadrants: (a) urgent and important, (b) important but not urgent, (c) urgent but not important, and (d) not urgent and not important. The model helps prioritize tasks and focus on the most critical ones.

Nursing time management skills are essential for delivering efficient and effective patient care. Poor time management skills can lead to decreased patient satisfaction, inefficient use of resources, and ultimately compromised patient care [14]. Therefore, this study aimed to investigate sociodemographic and institution-level factors associated with time-wasting behaviors among nurses in Palestine. Specifically, the research questions were whether time-wasting behaviors vary by nurses' age, gender, place of residence, and educational levels. Of interest were the effects of institutional-level factors such as type of healthcare institution (public versus private), facility size (hospital versus clinic), and teaching status (teaching versus nonteaching institutions) on time-wasting activities. The impact of attending a time-management course on time-wasting behaviors was also investigated.

## 2. Methods

The surveys were collected in the period between March and August 2019 from 714 nurses working in 17 hospitals and multiple primary health care clinics in the North of the West Bank of Palestine. Approval was obtained from the Palestinian Ministry of Health. The researcher contacted the nursing directors of each hospital and primary healthcare center, inquiring about the number of nurses employed within their respective organizations. Subsequently, the researcher prepared questionnaires based on the gathered information, met with each nursing director in person, delivered the questionnaires, and explained the data collection process, including its content, objectives, and the process of obtaining consent.

The nursing directors were then tasked with distributing the questionnaires to all nurses in their organizations and collecting the completed questionnaires within a week. The researcher personally collected the completed questionnaires from the nursing directors. All nurses completed the questionnaires except those who were sick or on leave.

Inclusion criteria: all male and female staff and practical nurses who were available at the time of the data collection. Exclusion criteria: nurses who were on a sabbatical leave, a sick leave, nurse volunteers, and internship student nurses. The entire process was completed within a span of six months, and the researcher, along with the assistance of two experienced data entry personnel, entered the data into SPSS version 25. The collected data were analyzed using *t*-tests and ANOVA. To account for confounding effects, multivariate linear regression was employed.

### 2.1. Study Instrument

The study examined various sociodemographic variables including gender, age, residence, educational level, and years of experience. Time-wasting behaviors were evaluated using an 11-item scale developed based on prior research [28]. This scale measures different aspects of time-wasting behaviors, such as excessive grooming, poor planning, failing to distinguish between important and unimportant tasks, underestimating task completion time, excessive use of social media, and excessive socialization. Responses were recorded on a 5-point scale ranging from “never = 1” to “always = 5” and a summative score was computed. Scores could range from a minimum of 11 to a maximum of 55, with higher scores indicating more frequent engagement in time-wasting activities.

The face and content validity of the study were ensured through consultations with 10 experts in nursing and time management. These experts were briefed on the study's objectives and the construct of the scale. They were then asked to assess whether the items clearly and effectively measured their intended concepts (face validity). Additionally, the experts provided feedback on the relevance of the items and their representation of the content domains within the intended construct.

The author carefully analyzed the feedback received from the experts and made necessary adjustments to the wording and number of items. Subsequently, the revised questionnaire was pilot-tested with a group of 5 staff nurses. Construct validity was evaluated through factor analysis, and reliability was assessed using Cronbach's alpha.

## 3. Results

Cronbach's alpha of the entire sample was 0.862, indicating good internal consistency. Factor analysis identified three distinct factors, as detailed in Table 1. The Kaiser–Meyer–Olkin (KMO) measure of sampling adequacy was 0.845, indicating strong partial correlations among the variables and justifying the use of factor analysis. Bartlett's test of sphericity returned a *p* value of less than 0.001, which rejects the null hypothesis that the variables are unrelated. This indicates that the correlation matrix is not an identity matrix, affirming the appropriateness of factor analysis. None of the items had a commonality value above 0.4, indicating the appropriateness of factor analysis for exploring the data. Overall, the three-factor structure accounted for a substantial 76.77% of the variance in the nursing time management scale.

The divergent validity of the scale was assessed by correlating it with another scale which is theoretically intended to measure good time management behaviors among nurses. The time nursing wasting scale correlated negatively with the nursing time managements scale (NTMS), correlation coefficient = −0.162, *p* < 0.001. The NTMS is a scale that measures positive time management skills among nurses such as planning, goal setting, and coordination of activity (Table 1).

The factor loadings, means, and standard deviations for each item within the time-wasting scale are displayed in Table 2. Higher mean values indicate more frequent engagement in time-wasting behaviors among the participants. Among the various behaviors, grooming appeared to be the least time-consuming activity, with a mean score of 2.14. In contrast, work interruptions, such as being overly accessible, ranked as the most significant time-wasting factor, with a mean score of 3.15. Following closely were behaviors like not having specific time to respond to emails and phone calls (mean of 3.03) and continuously checking emails and other communication media (mean of 2.83).

The factor analysis unveiled a three-factor model, categorizing time-wasting behaviors into three dimensions:

1. Inability to plan and organize tasks
2. Improper use of technology and social media
3. Excessive socialization

Table 3 shows the frequencies and means of the time-wasting scores by background variables. Of the 714 participants, 34.8% were male and 65.0% were female. The mean time-wasting score for male participants was 28.02, and for female participants, it was 27.38, *p*=0.223. The average time-wasting score of nurses over the age of 35 is higher than younger age groups, *p* < 0.001. The mean time-wasting score was highest for participants living in the camp (29.25) and lowest for those living in the city (27.00).

Regarding workplace, the table shows that the mean time-wasting score was significantly higher for participants working in a hospital (28.53) compared to those working in a patient community clinic (25.17) (*P* < 0.001). For this type of organization, the mean time-wasting score was significantly higher for participants working in a private organization (31.53) compared to those working in a government organization (26.31) (*P* < 0.001).

Regarding the current job position, the table shows that 631 participants (88.3%) were nurses, while 82 participants (11.5%) were nurse supervisors. The mean time-wasting score was similar across the two job positions, with no statistically significant difference (*P*=0.342). Participants who worked in teaching hospitals/clinics had a lower mean time-wasting score (26.59) than those who did not work in teaching hospitals/clinics (28.88), and those who attended a time management course had a slightly lower mean time-wasting score (27.00) than those who did not attend a time management course (28.44).

The multivariate linear regression in Table 4 shows no significant relationship between time wasting and gender, age, place of residence, educational level, and job experience. On the other hand, place of work emerged as a significant predictor of the time-wasting score when other background variables were considered. Individuals working in community clinics have lower time-wasting average scores compared to those working in hospitals (*B* = −3.2, *p* < 0.001).

The type of organization was also a significant predictor of time management skills. Individuals working in private organizations engaged in more time-wasting activities compared to those working in government organizations (*B* = 4.09, *p* < 0.001).

Nurses in nonteaching hospitals are more likely to engage in time-wasting activities than nurses in teaching hospitals (*B* = 2.63, *p* < 0.001). Nurses who had not attended a time management course obtained a higher score on the time-wasting scale compared to nurses who had attended a time management course (*B* = 3.11, *p* < 0.01).

## 4. Discussion

This research sought to examine the predictors of time-wasting behaviors among nurses. The results indicate that time-wasting behaviors are influenced by individual-level and organizational-level factors. The finding that attending a time management course is effective in reducing time-wasting activities and improving time management skills corroborates numerous previous studies that have provided evidence for the positive impact of time management training [19, 22, 29–31].

Prior studies have demonstrated that time-wasting activities decrease with age [22]. In the current study, older age was associated with lower time-wasting scores in the bivariate analysis, but this association did not hold in the multivariate analysis, most likely due to adjustments for confounding variables such as seniority or job experience. Similarly, job experience was associated with lower time-wasting scores in the bivariate analysis, but this relationship did not persist in the multivariate analysis. This finding contradicts previous research, which found significant correlations between years of experience and improved time management skills [23]. Educational level, however, was not associated with time-wasting behavior in this study. This result aligns with findings from a study in Bangladesh that failed to establish a relationship between educational level and time management skills [32]. In contrast, a study in Iran found significant positive correlations between time management skills and age, education, job experience, and managerial experience, probably because this study did not adjust for potential confounding effects [16].

Organizational types, such as hospital vs. community clinic or government vs. private, were strongly statistically related to time-wasting scores in this study. This suggests that organizational-level factors could influence time-management behaviors. Elsayed et al. [23] reported that organizational factors, such as understaffing, work overload due to a large number of visitors, and the absence of clear management plans, strongly influence employees' time management behaviors. Others have found that poor organizational and policy factors, such as frequent staff meetings, inefficient and unclear communications, and excessive administrative work, such as writing attendance records or tasks unrelated to their specialty, can result in significant time wastage among employees [33].

Time-wasting activities, as reported by nurses in this and previous studies, include spending too much time on low-priority tasks; treating everything as urgent; inability to say “no” to extra work; and excessive interruptions by people and colleagues [34]. Meetings that are ineffective, with no clear purpose, agenda, or follow-ups are a waste of time as well as arriving at meetings late. Other time-wasters include excessive socializing, phone calls, and phone interruptions; inefficient and unclear communications with patients and colleagues; insufficiently detailed policies and procedures; reluctance to delegate tasks; and an overload of paperwork [34].

**Figure 1:**
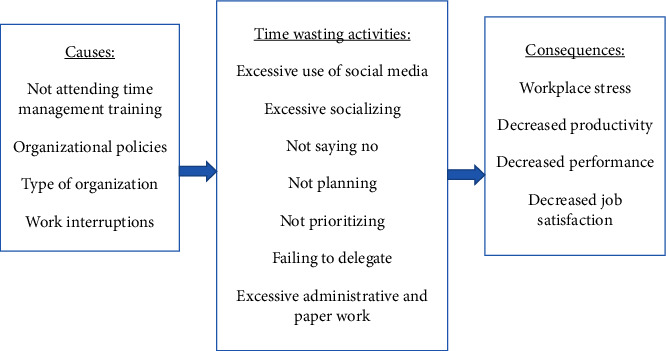
A theoretical framework of types, causes, and consequences of time-wasting behaviors.

Not knowing how to prioritize, plan, and handle interruptions can lead to increased errors and work inefficiencies [35, 36]. The findings from this study concur with previous research, which shows the inability of nurses to say “no” as one of the most important factors negatively influencing nurses' workflow [19, 35]. It is known that in Arab culture, saying “no” or “I do not know” is not commonly used, probably because such terms are thought of as impolite. Nevertheless, nurses should learn to refuse additional tasks that they cannot perform or tasks that interrupt their workflow. Other major time-wasters mentioned in previous research include not knowing how to delegate.  Figure 1 illustrates a theoretical framework of the types, causes, and consequences of time wasting in the nursing profession. The figure summarizes the findings from this article and previous studies.

### 4.1. Limitations

While this study was carried out on a representative sample within the northern regions of Palestine, it is important to note that generalizing the findings to other contexts may not be fully established. To strengthen the generalizability of the findings, it is necessary to conduct further research in various other contexts. Moreover, as a cross-sectional study, causal inferences are constrained. Nevertheless, one strength of this study lies in its high sample size, addressing the constraints associated with smaller sample sizes in earlier research.

Although having the nursing directors distribute and collect the surveys has increased response rates and motivation to complete the questionnaires with precision, it cannot be ruled out that some respondents may have been unduly influenced to participate despite the fact that it was explained to them that participation is voluntary.

## 5. Conclusions

According to the results of this research, providing time management courses to nurses can be an effective strategy to reduce time waste and improve work quality. However, in addition to providing time management courses, hospitals and clinics should also consider implementing organizational policies and strategies to reduce time-wasting activities.

### 5.1. Recommendations

Hospitals and clinics should conduct regular time management courses for their employees, with an emphasis on providing practical instructions and exercises on how to avoid and eliminate time-wasting activities such as interruptions, distractions, and procrastinations. In addition, organizations should design policies that minimize time-wasting activities, such as requiring excessive reporting, vague communications, and unnecessary meetings. Such policies should be clearly communicated to all employees.

In addition to time management courses, organizations should also provide their employees with the necessary tools and resources to manage their time effectively, such as time-tracking software or prioritization frameworks. By following these recommendations, hospitals and clinics can create a more productive work environment and improve the quality of care they provide to their patients.

## Figures and Tables

**Table 1:** Correlation between the time-wasting scale and NTMS.

		NTMS	Time-wasting scale
NTMS	Pearson correlation	1	
	Sig. (2-tailed)		

Time-wasting scale	Pearson correlation	−0.162^*∗∗*^	1
	Sig. (2-tailed)	0.000	

^∗^ *p* < 0.05; ^∗∗^*p* < 0.01; ^∗∗∗^*p* < 0.001.

**Table 2:** Factor loading, means, and SD for each question in the time-wasting scale.

	1	2	3	Mean	SD
You spend more time with personal grooming than doing nursing work	0.81			2.14	1.30
You do not understand time planning	0.89			2.24	1.38
You cannot distinguish what is important from what is not	0.90			2.22	1.39
You underestimate time and effort needed to accomplish tasks	0.89			2.23	1.37
You are on Internet access all the time during the day		0.75		2.62	1.37
You check and respond to so many different communication mediums (e-mail, voice mail, others)		0.79		2.83	1.37
You have assigned times to respond to phone calls or emails, reversed		0.73		3.03	1.33
You make yourself overly accessible		0.76		3.15	1.37
When someone is talking and not getting to the point, you interrupt him and search for truth			0.84	2.37	1.19
You are brief when talking, reversed			0.88	2.37	1.17
When you are busy, you give appointments when you are free, reversed			0.84	2.41	1.16

**Table 3:** Time-wasting scores by background variables, *N* = 714.

	*n*	%	Mean	SD	*P*
*Gender*					
Male	249	34.8	28.02	7.02	0.223
Female	465	65.0	27.38	6.42	

*Age*					
Less than	201	28.1	28.16	6.69	**≤0.001**
25–35	200	28.0	28.79	7.20	
More than 35	313	43.8	26.50	6.05	

*Residence*					
City	352	49.2	27.00	6.54	**0.020**
Village	299	41.8	27.98	6.64	
Camp	63	8.8	29.25	6.86	

*Educational level*					
Technical	271	37.9	27.66	6.87	0.973
Bachelor	409	57.2	27.58	6.51	
Master or above	34	4.8	27.41	6.39	

*Workplace*					
Hospital	518	72.4	28.53	6.96	**≤0.001**
Patient community clinic	196	27.4	25.17	4.94	

*Type of organization*					
Government	537	75.1	26.31	6.51	**≤0.001**
Private	177	24.8	31.53	5.36	

*Current job position*					
Nurse	631	88.3	27.53	6.59	0.342
Nurse supervisor	82	11.5	28.27	6.99	

*Job experience*					
Less than 5 years	225	31.5	28.24	6.65	**0.003**
Between 5 10 years	165	23.1	28.55	7.09	
More than 10 years	324	45.3	26.69	6.28	

*Teaching hospital/clinic*					
Yes	396	55.4	26.59	6.93	**≤0.001**
No	318	44.5	28.88	6.01	

*Attended time management course*					
Yes	415	58.0	27.00	6.88	**0.004**
No	299	41.8	28.44	6.19	

*Total*	714				

The bold values are statistically significant. Note. ^∗^*p* < 0.05; ^∗∗^*p* < 0.01; ^∗∗∗^*p* < 0.001.

**Table 4:** Multivariate linear regression of time-wasting score by background variables, *N* = 714.

	*B*	*P*	95% CI	
*Gender*				
Male	ref			
Female	0.44	0.392	−0.573	1.460

*Age*				
Less than 25	ref			
25–35	1.00	0.115	−0.245	2.244
More than 35	0.52	0.478	−1.976	0.926

*Residence*				
City	ref			
Village	0.53	0.274	−0.423	1.490
Camp	1.05	0.209	−0.591	2.692

*Educational level*				
Technical	ref			
Bachelor	0.76	0.129	−0.222	1.734
Master or above	0.73	0.526	−1.528	2.988

*Workplace*				
Hospital	ref			
Patient community clinic	3.02	**≤0.001**	−4.360	−1.675

*Type of organization*				
Government	ref			
Private	4.09	**≤0.001**	2.925	5.246

*Current job position*				
Nurse	ref			
Nurse supervisor	0.74	0.340	−0.785	2.273

*Job experience*				
Less than 5 years	ref			
Between 5 10 years	0.20	0.761	−1.082	1.478
More than 10 years	0.34	0.638	−1.085	1.768

*Teaching hospital/clinic*				
Yes	ref			
No	2.63	**≤0.001**	1.644	3.626

*Attended time management course*				
Yes	ref			
No	3.11	**0.002**	0.576	2.556

The bold values are statistically significant. Note. ^∗^*p* < 0.05; ^∗∗^*p* < 0.01; ^∗∗∗^*p* < 0.001.

## Data Availability

The dataset examined in the present study can be obtained from the corresponding author upon a reasonable request.
